# Editorial commentary for the special issue: technological developments in hyperpolarized ^13^C imaging—toward a deeper understanding of tumor metabolism in vivo

**DOI:** 10.1007/s10334-021-00908-1

**Published:** 2021-02-13

**Authors:** Kevin M. Brindle, Kayvan R. Keshari

**Affiliations:** 1 Cambridge, UK; 2 New York, USA

Dynamic nuclear spin polarization (DNP), in which polarization is transferred from electron to nuclear spins at low temperature and high magnetic field, has a long history, with the first experiments being performed in the 1950s [1]. However, it was not until 2003, when it was shown that molecules containing hyperpolarized ^13^C spins at ~ 1.2 K could be rapidly warmed to room temperature, with substantial retention of this nuclear spin polarization [2], that the power of this technology was unleashed for solution state NMR studies. A major focus has been on metabolic imaging with hyperpolarized ^13^C-labeled cell substrates, where the > 10^4^ gain in sensitivity has enabled ^13^C magnetic resonance spectroscopic (MRSI) imaging of injected hyperpolarized ^13^C-labelled substrates (probes) in the body and their biochemical conversion to metabolic products. Basic and preclinical research leveraging these hyperpolarized probes (reviewed in [3–6]), culminated in translation of this approach to the clinic with a study of hyperpolarized [1-^13^C]pyruvate metabolism in prostate cancer [7]. The technique now stands on the threshold of wider clinical application with the publication of further studies in prostate cancer [8–12], and initial studies of hyperpolarized [1-^13^C]pyruvate metabolism in brain [13, 14] and heart tissue [15, 16] and in tumors of the brain [17–20], breast [21, 22], pancreas [23] and kidney [24]. An alternative approach to hyperpolarizing ^13^C spins, which is reviewed in this issue, is to transfer spin order from parahydrogen. The advantage of this method is that the equipment is less expensive and polarization times are much shorter, allowing for semi-continuous production. However, the levels of polarization achieved in molecules of immediate interest, such as pyruvate, are much lower and although [1-^13^C]pyruvate polarized using this method has been used in vivo the technique is at a much earlier stage on the path to clinical translation.

[1-^13^C]pyruvate has been the most widely used substrate as it can be polarized to high levels (> 60% at 7T [25]) with sufficiently long ^13^C T_1_ relaxation times in vivo to facilitate metabolic imaging. The door is now open to pursue many new probes but as the field is consolidating, it faces 2 major challenges: (1) What is the role of this imaging modality in the management of disease? (2) Can some of the technical hurdles facing hyperpolarized MRI be overcome in order to facilitate widespread use of this approach.

1. Preclinical and recent clinical studies have suggested that hyperpolarized metabolic imaging can provide a non-radioactive approach to assess tumor grade, progression and treatment response [8, 11, 12, 19, 22]. Beyond cancer, this has further been exemplified in highly metabolic organs including studies in the brain and heart, where understanding disease can be unlocked by flux measurements using HP MRI. The technique has not the breadth of nuclear medicine imaging, but then this has a history of more than 50 years in patients. In order to progress the field, many more studies are required to truly say something relevant about human biology, though current technical limitations preclude such studies from being achieved.
2. While high polarizations have been achieved for substrates at high concentration, current approaches are limited due to low throughput, long polarization times and challenges with respect to final concentrations. New developments highlighted in this special issue provide dramatic improvements in both polarization methodologies and the ability to separate polarization from dissolution. These strategies can reduce the technical burdens of hyperpolarization and could facilitate its application to clinical studies. Further, many imaging approaches have been designed to encode these rapidly decaying and interconverting signals. Uniformity across platforms is required to be able to compare such data. In an effort to get closer to consensus, reviews from experts in hyperpolarized imaging are needed which compare the strengths and weaknesses of these imaging strategies.

While these technical hurdles must be overcome to truly address the needs of the field, the future is encouraging and new developments are paving the way to a more holistic view of metabolism in vivo.
